# Supplementary material for: Life events and adolescent depressive symptoms: Protective factors associated with resilience
Source: PLoS One. 2020 Jun 5;15(6):e0234109. doi: 10.1371/journal.pone.0234109 (PMC7274383; doi:10.1371/journal.pone.0234109)
Supplement: S1 Table — Abbreviated wording of items adapted from Von Soest et al., 2010. (DOCX) [file pone.0234109.s001.docx]

| Item |  |
| --- | --- |
| *Goal Orientation* | |
| 1 | Goal orientation |
| 2 | Aims and objectives |
| 7 | Know how to reach goals |
| 8 | Planfulness |
| 13 | Organizational skills |
|  |  |
| *Self-Confidence* | |
| 17 | Feeling competent |
| 20 | Confident in making the right choices |
| 23 | Believe in myself |
| 26 | Positive outlook despite hardship |
|  |  |
| *Social Competence* | |
| 6 | Positive social orientation |
| 11 | Easily make friends |
| 16 | Good at talking to new people |
| 22 | Find fun conversation topics |
|  |  |
| *Social Support* | |
| 3 | Encouragement from friends/family |
| 14 | Friends/family cares |
| 19 | Have someone who can help |
| 28 | Appreciated by friends/family |
|  |  |
| *Family Cohesion* | |
| 5 | Shared values in family |
| 10 | Comfortable with family |
| 15 | Agreement in family |
| 18 | Rules in family |
| 21 | Common positive outlook in family |
| 24 | Family support |
| 27 | Shared activity in family |
